# Global soil respiration estimation based on ecological big data and machine learning model

**DOI:** 10.1038/s41598-024-64235-w

**Published:** 2024-06-09

**Authors:** Jiangnan Liu, Junguo Hu, Haoqi Liu, Kanglai Han

**Affiliations:** 1 College of Chemistry and Materials Engineering, Zhejiang A&F University, Hangzhou, 310000 China; 2 College of Electrical and Information Engineering, Quzhou University, Quzhou, 324000 China; 3 College of Mathematics and Computer Science, Zhejiang A&F University, Hangzhou, 310000 China

**Keywords:** Global soil respiration, Machine learning, Random forest, Ecological big data, Environmental impact, Scientific data

## Abstract

Soil respiration (Rs) represents the greatest carbon dioxide flux from terrestrial ecosystems to the atmosphere. However, its environmental drivers are not fully understood, and there are still significant uncertainties in soil respiration model estimates. This study aimed to estimate the spatial distribution pattern and driving mechanism of global soil respiration by constructing a machine learning model method based on ecological big data. First, we constructed ecological big data containing five categories of 27-dimensional environmental factors. We then used four typical machine learning methods to develop the performance of machine learning models under four training strategies and explored the relationship between soil respiration and environmental factors. Finally, we used the RF machine learning algorithm to estimate the global Rs spatial distribution pattern in 2021, driven by multiple dimensions of environmental factors, and derived the annual soil respiration values. The results showed that RF performed better under the four training strategies, with a coefficient of determination R^2^ = 0.78216, root mean squared error (RMSE) = 285.8964 gCm^−2^y^−1^, and mean absolute error (MAE) = 180.4186 gCm^−2^y^−1^, which was more suitable for the estimation of large-scale soil respiration. In terms of the importance of environmental factors, unlike previous studies, we found that the influence of geographical location was greater than that of MAP. Another new finding was that enhanced vegetation index 2 (EVI2) had a higher contribution to soil respiration estimates than the enhanced vegetation index (EVI) and normalized vegetation index (NDVI). Our results confirm the potential of utilizing ecological big data for spatially large-scale Rs estimations. Ecological big data and machine learning algorithms can be considered to improve the spatial distribution patterns and driver analysis of Rs.

## Introduction

Significant uncertainties remain in the current global carbon budget and terrestrial carbon cycle. As an essential part of the terrestrial carbon cycle, soil respiration connects atmospheric and subsurface carbon^1–8^. Rs is the maximum carbon dioxide flux from terrestrial ecosystems to the atmosphere. However, owing to insufficient consideration of the impact of environmental factors and improper model selection, there are still significant uncertainties in the estimation of soil respiration, and the relationship between soil respiration and environmental factors remains unclear^9–12^. Generally, the estimation models for soil respiration are divided into process and empirical statistical models. Process-based soil respiration models (e.g., BEPS^13^, CLASS-CTEM^14^, CLM5.0^15^, CABLE^16^, IBIS^17^, LPX-Bern^18^, LPJ-GUSS^19,20^, ORCHIDEE^21^, VISIT^22^, and VEGAS^23^) explore the interaction between soil respiration intensity and environmental conditions by simulating the soil respiration formation process and further assessing the temporal and spatial variation in RS on a regional scale^24,25^. However, it is usually time-consuming to estimate soil respiration on a large scale in time and space with a process model^26^. In addition, the compromise between model complexity and calculation timeliness inevitably reduces the accuracy of estimation results^27^. Finally, different process models consider different environmental factors and structures, resulting in differences in the regional soil respiration estimates^28^.

Compared with the process model, the statistical model does not rely on complex theories and assumptions but extracts the functional relationship according to the observation data to describe the nonlinear relationship between Rs and environmental variables^24^. Some studies have used traditional regression models to estimate Rs^9,29,30^. However, the relationship between soil respiration and environmental variables is often complex and nonlinear, and traditional statistical models often exhibit a low prediction accuracy when characterizing this nonlinear relationship.

Machine learning (ML) is an advanced statistical model that automates the analysis of modelling data. ML efficiently completed the regression task by learning to fit the driving relationship between soil respiration and the environmental variables. In recent years, an increasing number of soil respiration models have been established based on machine-learning models. At the site scale, Dou et al. used geographic location, elevation, and climate data to estimate daily carbon fluxes at eight FLUXNET sites based on artificial neural networks (ANN), support vector machines (SVM), adaptive neuro-fuzzy inference systems (ANFIS), and extreme learning machines(ELM) and evaluated the impact of model parameters^11^. Hanoon et al. used a gradient boosting tree (GBT), RF, linear regression (LR), and ANN to predict air temperature and humidity and evaluated the performance of the algorithm^31^. Hu et al. improved the estimates of the spatial distribution of soil respiration by using a Bayesian maximum entropy algorithm and soil temperature as auxiliary data^32^. However, the uncertainty in the estimation of soil respiration at the site scale is high because of the limited number of sampling sites and lack of large-scale and long-term observation data.

At the regional scale, Ebrahimi et al. used LR and ANN methods combined with soil sampling data, MAP, and MAT to estimate soil respiration, and the results showed that artificial neural networks could be used for soil respiration estimation^30^. However, considering that environmental factors include only a limited number of aspects, uncertainty still exists in the estimated results.

On a global scale, Jian et al. investigated the contribution of root respiration to soil respiration in the SRDB using the RF algorithm, but did not reach clear conclusions because of spatial variability in soil respiration^33^. Huang et al. used SVM, RF, and ANN machine learning methods to explore the relationship between the temporal-spatial variability of global soil respiration and land-use change, obtaining soil respiration data and driving factors^12^. Tramontana et al. used the RF algorithm to estimate GPP on three scales by combining meteorological and remote sensing data^34^. The selection of models in previous studies did not fully consider the typical types of machine learning and did not analyze the effects of environmental factors on Rs in five dimensions: temporal and spatial distribution factors, vegetation factors, soil property factors, meteorological factors, and land-use factors^35–37^.

The integration of multi-source ecological data for accurate estimation of soil respiration is crucial for clarifying terrestrial carbon cycle processes and addressing climate change^11^. Environmental variables from multiple sources and dimensions were used to estimate and predict soil respiration to improve estimation accuracy. For example, Zhu et al. used vegetation type, elevation, air temperature, land surface water index (LSWI), photosynthetically active radiation (PAR), normalized vegetation index (NDVI), enhanced vegetation index (EVI), and soil organic carbon density (SOCD) data as environmental variables to estimate ecosystem respiration using machine-learning methods. It was concluded that the enhanced vegetation index EVI is essential for improving model performance^37^. Warner et al. confirmed the contribution of meteorological variables such as MAT and MAP to soil respiration while estimating global soil respiration and uncertainty^35^. Other environmental factors included the physical and chemical properties of the soil, geospatial location distribution, and land-use^12,30,38^. In previous studies, to reduce the computational process time and hardware cost, a few of these variables were selected as the final model inputs through variable selections such as principal component analysis and importance ranking. The data dimensions used for soil respiration estimation were still not abundant, which may be one of the reasons for the lack of accuracy of the estimation results.

Soil respiration is comprised of both autotrophic and heterotrophic processes. It involves a variety of physical, chemical, and biological processes, and their interactions; therefore, soil respiration is susceptible to various environmental factors^25^. Temperature and precipitation are the most critical environmental factors affecting the strength of soil respiration^9,29,31^. In addition, RS is influenced by biomass, phenology, soil organic carbon content, soil physical and chemical properties (e.g., soil texture and pH), soil nutrient status (e.g., soil N and P content), and human activities (e.g., land-use change and grazing)^12,33,37,38^. Few studies have comprehensively considered the effects of climate, soil, vegetation, spatial and temporal, and land-use factors on the spatial and temporal variations in global soil respiration.

In response to the problems mentioned above in previous studies, this study hopes to use data science and machine learning methods to estimate soil respiration on a global scale. This study aimed to (1) investigate the performance of four typical machine learning models in estimating soil respiration using four training strategies; (2) quantify the contribution of multi-dimensional environmental factors to annual soil respiration; (3) explore the impact of human management in ecosystems on soil respiration; and (4) predict global soil respiration distribution patterns.

## Data and methodology

### Data

#### Soil respiration observation data

**Figure 1:**
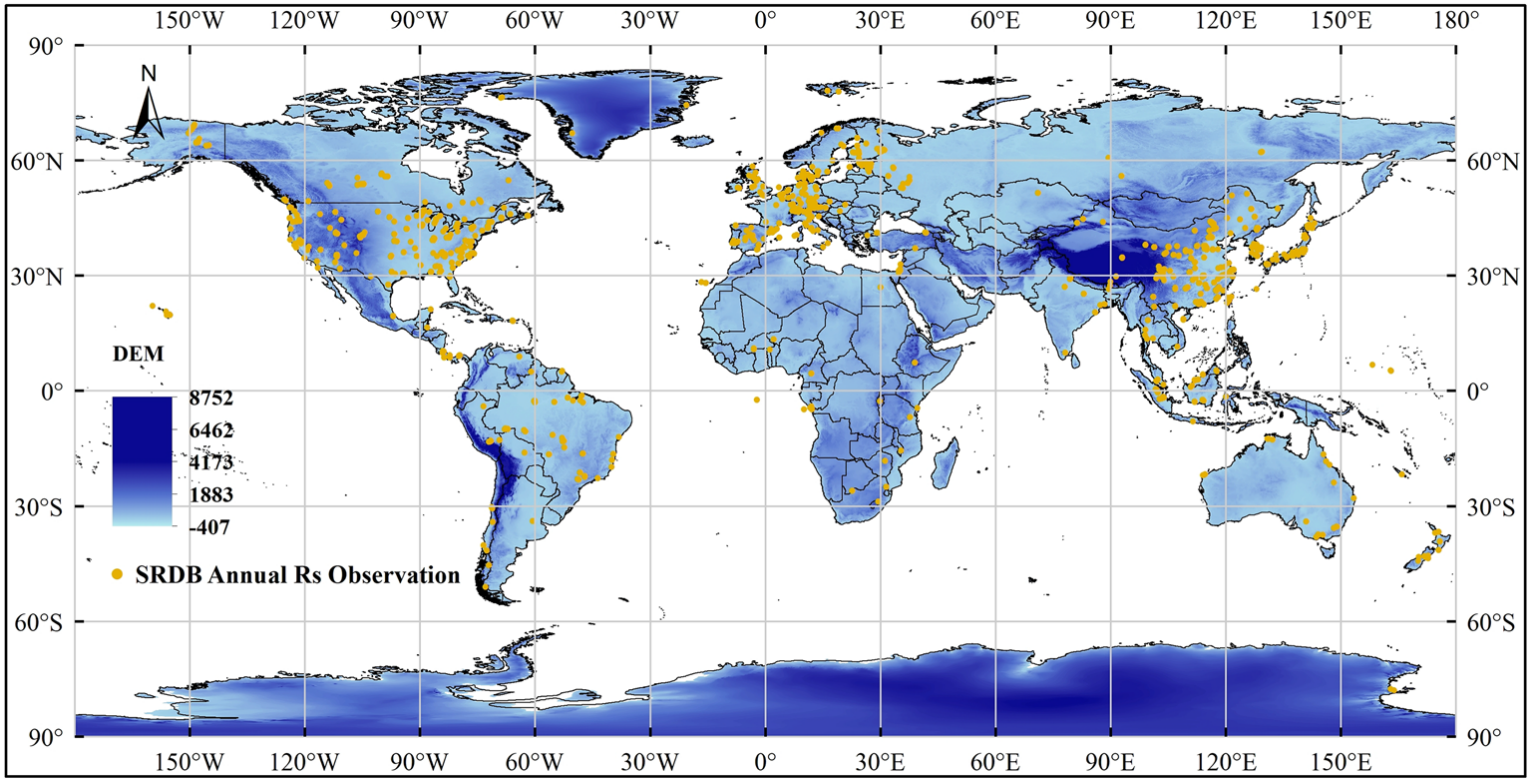
Distribution of 4888 annual soil respiration observations (yellow dots) on a global digital elevation map (blue shading), and the unit of elevation is meters. This image was created using ArcGIS, version 10.8.1, available at: https://www.arcgis.com.

The annual soil respiration observational data used in our study were obtained from the SRDB-V5^39^. SRDB-V5 compiled and organized published soil respiration observation data worldwide and compiled 10,839 soil respiration data (https://github.com/bpbond/srdb). We used the following data screening criteria to ensure the quality of the data: (i) we only selected data measured by an infrared gas analyzer (IRGA) and gas chromatography. (ii) focus on screening the annual Rs value and non-seasonal or shorter time period measurement results. (iii) Data marked in the “Quality_flag” field that may have problems are excluded. (iv) Low-quality data that are not universal, such as those in special scenarios, are excluded. After performing a series of data science operations, such as missing value processing, outlier processing, data normalization, and logarithmic transformation, we obtained 4888 soil respiration data records that met the criteria for screening over 33 years from 1986 to 2018. The distribution of the 4888 soil respiration observations is shown in Fig. 1.

#### Spatio-temporal data

The spatiotemporal data included the year of the study, corresponding to the soil respiration data, latitude and longitude data of the observation site, digital elevation data, and the type of climate zone in which the observation site was located. The study year and geographic location data (latitude and longitude) were obtained from the SRDB-V5. Digital elevation data were obtained by querying the corresponding elevation data from NASADEM line-by-line using latitude and longitude information^40^. Climate-type data for each observation site were obtained from the global Köppen-Geiger climate classification map^41^. Table 1 presents the details of the data.

**Table 1:** Environmental variables used to estimate soil respiration during 1986–2018.

Type	Term	Definition	Variable types	Data source
Spatial–Temporal Data	Study year	Years corresponding to soil respiration observations	Numeric	SRDB-V5
	Longitude	Longitude of the observation site	Numeric	SRDB-V5
	Latitude	Latitude of the observation site	Numeric	SRDB-V5
	Biome	Type of climate zone	Categorical	World Map of the climate classification updated
	Elevation	Digital Elevation of the Observation Site	Numeric	NASADEM^40^
Climate Data	MAT	Mean annual temperatures	Numeric	ERA5 Monthly Aggregates
	MAP	Mean annual precipitation	Numeric	ERA5 Monthly Aggregates
Vegetation Data	NDVI	Normalized Difference Vegetation Index	Numeric	Calculated based on the USGS Landsat datasets
	EVI	Enhance Vegetation Index	Numeric	Calculated based on the USGS Landsat datasets
	EVI2	Enhance Vegetation Index 2	Numeric	Calculated based on the USGS Landsat datasets
	Leaf habit	Dominant leaf habit (deciduous, evergreen, mixed)	Categorical	SRDB-V5
	Stage	Developmental stage (aggrading, mature)	Categorical	SRDB-V5
Soil Data	st_0_7cm	Soil temperature of soil layer 0 to 7 cm below the ground	Numeric	ERA5-Land Monthly Averaged
	st_7_28cm	Soil temperature in the soil layer 7 to 28 cm below the ground	Numeric	ERA5-Land Monthly Averaged
	sw_0_7cm	Soil water in the soil layer 0 to 7 cm below the ground	Numeric	ERA5-Land Monthly Averaged
	sw_7_28cm	Soil water in the soil layer 7 to 28 cm below the ground	Numeric	ERA5-Land Monthly Averaged
	SOC_0cm	Soil organic carbon content at 0 cm depth	Numeric	OpenLandMap Soil Organic Carbon Content
	SOC_10cm	Soil organic carbon content at 10 cm depth	Numeric	OpenLandMap Soil Organic Carbon Content
	SOC_30cm	Soil organic carbon content at 30 cm depth	Numeric	OpenLandMap Soil Organic Carbon Content
	SBD	Soil bulk density at 0 cm depth	Numeric	OpenLandMap Soil Bulk Density
	Soil pH	Soil pH in H_2_O at 0 cm depth	Numeric	OpenLandMap Soil pH in H2O
	Sand content	Sand content at 0 cm depth	Numeric	OpenLandMap Sand Content
	Clay content	Clay content at 0 cm depth	Numeric	OpenLandMap Clay Content
	Soil texture	Soil texture class at 0 cm depth	Categorical	OpenLandMap Soil Texture Class
	Soil drainage	Soil drainage (dry, wet, medium)	Categorical	SRDB-V5
Land-use Data	Ecosystem type	Land-use type (grassland, forest, etc.)	Categorical	SRDB-V5
	Ecosystem state	Land-use state (Managed, Unmanaged, Natural). 'Unmanaged' means human management or disturbance in the past, but not currently	Categorical	SRDB-V5

#### Climate data

Climate-related environmental variables were derived from the ERA5-Land monthly averaged data, including MAT and MAP. Based on the year of study and the latitude and longitude coordinates of the observation sites, MAT and MAP data were obtained by averaging the raster data corresponding to the observation sites monthly using the Google Earth Engine (GEE) platform^42^.

#### Soil data

The soil properties play a crucial role in soil respiration. Nine soil properties were selected as predictors: temperature (ST), moisture (SW), organic carbon (SOC), bulk density (SBD), pH, sand content, clay content, texture class, and drainage. Soil properties vary at different depths and may affect soil respiration. Soil temperature and water were chosen from the ERA5-Land monthly averaged data at depths of 0–7 cm and 7–28 cm^43^. Soil organic carbon was extracted from OpenLandMap (https://openlandmap.org) at depths of 0, 10, and 30 cm. In summary, 13 soil-related environmental predictors were identified in this study.

#### Vegetation data

Different types of vegetation index data are widely used to predict and estimate soil respiration. For example, Warner et al. considered four predictors, such as EVI, and used a quantile regression forest to estimate Rs^35^. Huang et al. used EVI to analyze spatiotemporal variations in soil respiration^12^. Zhu et al. estimated the ecosystem respiration of grasslands by considering NDVI and EVI^37^. Previous studies have usually used NDVI, EVI, or their combination as the vegetation index to estimate Rs and concluded that the vegetation index plays an important role in improving the estimation results of soil respiration. In addition, EVI2 is considered to be an enhancement of EVI and has been used in fields such as predicting crop yield in wheat^44^. However, EVI2 has not been applied to estimate soil respiration, and it is unclear whether it can improve the estimation of Rs. Therefore, in this study, three vegetation indices, NDVI, EVI, and EVI2, were used to estimate the global soil respiration. USGS Landsat datasets were used to calculate NDVI, EVI, and EVI2 data on the Google Earth Engine (GEE) platform for the corresponding study years and locations^45^. The calculation formulas for NDVI, EVI, and EVI2 are shown in Eqs. (1), (2), and (3), respectively^46^. where N, R, and B are the atmospherically corrected surface reflectances for the near-infrared, red, and blue bands, respectively.

1 $$NDVI=\frac{N-R}{N+R}$$

2 $$EVI=2.5\frac{N-R}{N+6R-7.5B+1}$$

3 $$EVI2= 2.5\frac{N-R}{N+2.4R+1}$$

Because the soil respiration data spanned 33 years (from 1986 to 2018), vegetation indices could not be calculated using a particular data product. On balance, we decided that the vegetation indices corresponding to soil respiration for 1984–1998 were calculated using the Landsat 5 data product, those for 1999–2013 were calculated using the Landsat 7 data product, and those for 2014–2018 were calculated using the more updated Landsat 8.

Leaf habit refers to dominant vegetation as a categorical variable, including evergreen, deciduous, and mixed vegetation. Stage is also a categorical variable that refers to whether vegetation is growing or mature. Leaf habit and stage data were obtained from the SRDB.

#### Land-use data

Land-use data included the type of land-use and land-use state of the observation site. Land-use type refers to the vegetation cover of the observation site, such as forests and grasslands. Land-use refers to the presence of artificial disturbances in ecosystem development. It is a categorical variable divided into natural, managed, and unmanaged. Natural refers to an ecosystem that has not been disturbed by humans, managed refers to an ecosystem undergoing human intervention, and unmanaged refers to an ecosystem that has been managed but is not currently.

### Modelling methodology

#### Environmental variables

In this study, we not only synthesized the environmental factors involved in previous studies, but also added environmental factors that may be related to soil respiration, a total of 27 environmental factors. These variables covered five categories: time, space, climate, vegetation, soil, and land-use, and the selected variables were comprehensive.

**Figure 2:**
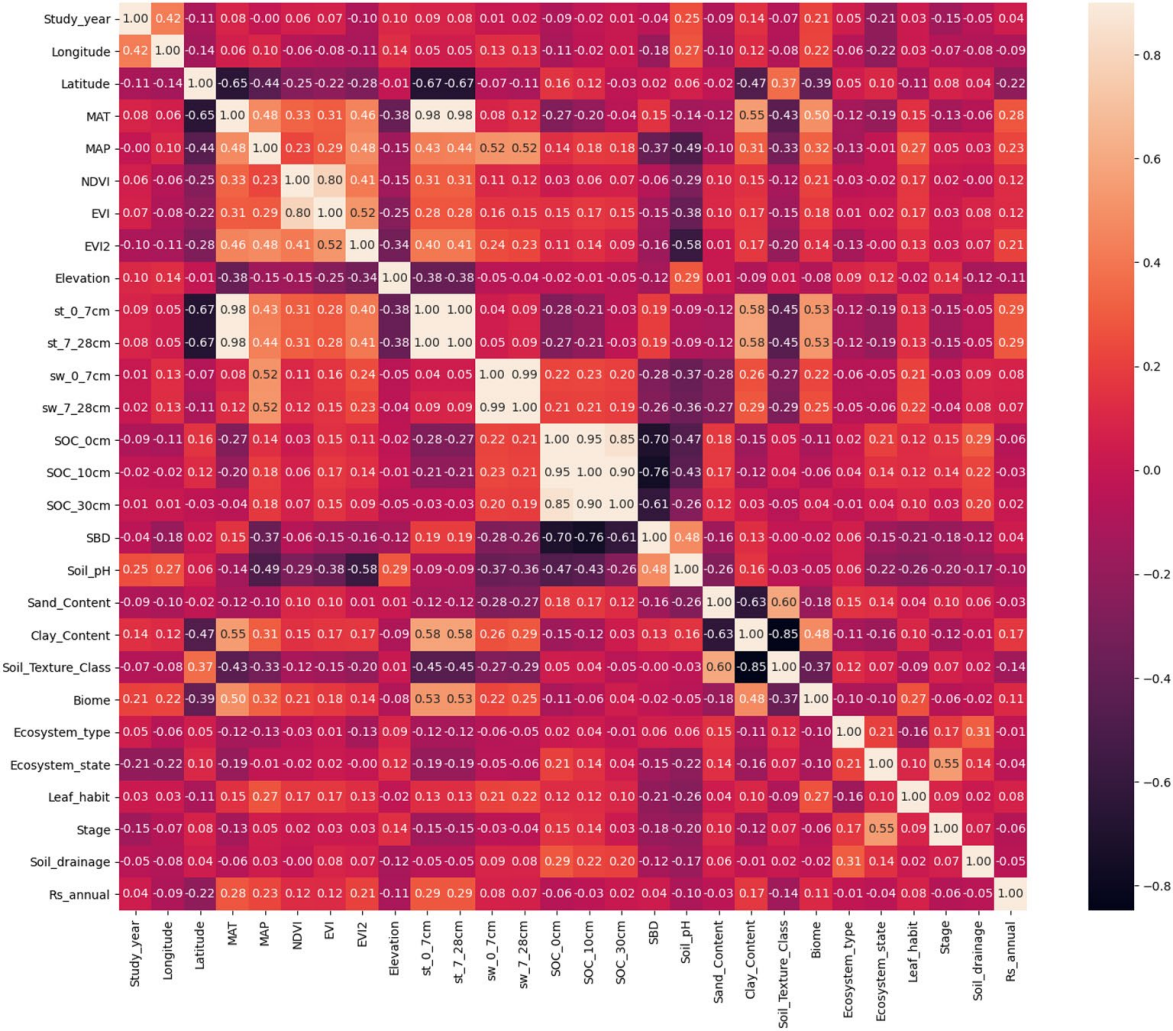
Pearson's standard correlation coefficient matrix between pairs of input variables.

The correlation of the input variables affects the machine learning algorithm used to resolve the causal relationship. Therefore, a linear correlation analysis was performed on the input variables pair by pair. By calculating the Pearson standard correlation coefficient matrix between variables (Fig. 2), we found that (i) latitude was negatively correlated with MAT; that is, MAT decreased with an increase in latitude; (ii) NDVI and EVI showed a strong positive correlation; (iii) soil temperature showed a strong positive correlation with MAT; (iv) soil moisture at different depths showed a positive correlation, so only one was maintained; (v) SOC and SBD at different depths showed a strong negative correlation; (vi) there was a strong negative correlation between clay content and sand content, and a strong negative correlation between clay content and soil texture class, where the soil sand content was retained.

Based on the above analysis results, we selected 17 variable characteristics: the study year, longitude, MAT, MAP, EVI, EVI2, Elevation, 0 cm soil water content, 0 cm soil organic carbon, soil pH, sand content, biome, ecosystem type, ecosystem state, leaf habit, stage, and soil drainage as inputs to the model.

#### Machine learning algorithms

As a type of feedforward neural network, an MLP typically models three network layers: input layer, hidden layer, and output layer, where the hidden layer can have multiple layers. In this study, a multi-layer perceptron regressor with a hidden layer of 1000 neurons, a “relu” hidden layer activation function, and an “lbfgs” weight optimization solver was used. alpha = 0.015 for the L2 penalty (regular term). The learning rate of the weight update was 0.001, and the optimization tolerance was set to 0.0001.

SVR is the application of a support vector machine (SVM) to regression problems. One of the main advantages of SVR is that its computational complexity does not depend on the input space dimensionality. In addition, it has an excellent generalization ability and high prediction accuracy. We set the penalty factor of the error term in the SVR model to 20 and choose “rbf” as the kernel type to train the multi-dimensional environment factor.

The GBDT is an additive model based on the idea of boosting integrated learning. During training, a forward distribution algorithm is used for greedy learning, and each iteration learns a classification and regression tree (CART) to fit the residuals between the previous predictions and the actual values of the training samples. The GBDT model is characterized by easy feature combination and selection, high prediction accuracy, and flexibility in handling continuous and dispersed data. The model learns and fits the nonlinear relationship between soil respiration and environmental variables to assess the importance of environmental factors under parameter settings of 1000 estimators, learning rate of 0.1, and maximum depth of 40 for a single regressor.

The RF is a typical bagging algorithm used in integrated learning. It is a combination of many decision trees and random means of randomly sampling from the dataset to train each decision tree in the model. Different datasets were randomly selected to ensure that each decision tree handled the problem from a different perspective, intending to output similar, but not identical, model results. The results for all decision trees were combined as the outputs. This training approach makes it difficult to overfit and insensitive to noise. RF has the features of high prediction accuracy, less susceptibility to overfitting, strong resistance to noise, ability to handle high-dimensional data with more features, multiple decision trees independent of each other, and time saving. In our study, we set the number of estimators of the model to 1000 and the maximum depth of the tree to 40 to determine the relationship between global soil respiration and environmental variables. Based on this, the performance of the model and the importance of the environmental variables were evaluated.

#### Model training strategy and development

**Figure 3:**
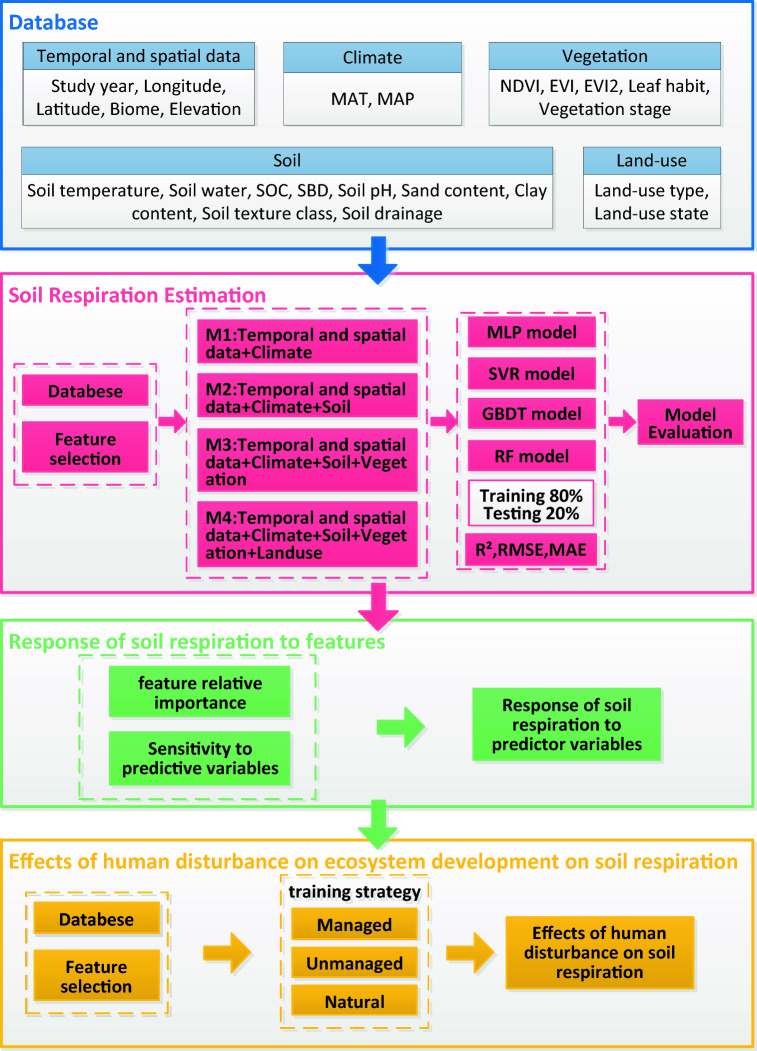
A framework for global soil respiration estimation based on multi-dimensional environmental variables and machine learning methods. *MAT* Mean Annual Temperatures, *MAP* Mean Annual Precipitation, *EVI* Enhance Vegetation Index, *EVI2* Enhance Vegetation Index 2, *NDVI* Normalized Difference Vegetation Index, *SOC* Soil organic carbon content, *SBD* Soil bulk density.

The general framework of this study is shown in Fig. 3. The input predictors were classified into five categories, and four training strategies were used to predict and estimate the soil respiration. The performance of four typical machine learning models and the importance of environmental factors were evaluated.

The estimates and drivers of soil respiration remain largely uncertain. Therefore, we integrated five types of data to constitute ecological big data and explored the response of soil respiration to the 17 predictors under four training strategies. To better analyze the impact of environmental variables on soil respiration, we used four strategies to train, optimize, and improve the model. According to the change in input variables, M1 used spatiotemporal and climate data; M2 used spatiotemporal, climate, and soil data; M3 used spatiotemporal, climate, soil, and vegetation data; and M4 used spatiotemporal, climate, soil, vegetation, and land-use data. Details of the training strategies are presented in Table 2.

**Table 2:** Training strategy.

Training strategy	Types of environmental factors	Number of environment variables
M1	Spatio-temporal and climate data	6
M2	Spatio-temporal, climate, and soil data	11
M3	Spatio-temporal, climate, soil, and vegetation data	15
M4	Spatiotemporal, climate, soil, vegetation, and land-use data	17

#### Model evaluation

The performance of the four machine learning models (MLP, SVR, GBDT, and RF) in estimating soil respiration was evaluated using a tenfold cross-validation strategy and training, validation, and testing of the model in this manner. First, we integrated the multi-source data and constructed a soil respiration dataset. To evaluate the model, we used a tenfold cross-validation method. tenfold cross-validation is a dynamic validation method that reduces the impact of data division. The specific steps are as follows. (1) We divided the dataset into a training set and a test set, placed the test set aside, and maintained the independence of the test set. (2) The training set was divided into ten parts. (3) One of the ten parts was used as the validation set each time, and all the others were used as the training set. (4) Ten different models were obtained after ten training sessions. (5) Evaluate the effects of the ten models and select the best hyperparameters. (6) Use the optimal hyperparameters and then retrain the model using all ten parts of the data as training sets to obtain the final model. The test set accounted for 20% of the data. It has been independent since the beginning of model training, and has never participated in the training and verification of the model. The parameters in the four models were optimized and determined using the grid search method^47^. The predicted values were compared with actual observed values. We chose the coefficient of determination (R^2^), root mean square error (RMSE), and mean absolute error (MAE) as the evaluation metrics to evaluate the model. These metrics were calculated as follows:

4 $${R}^{2}={\left(\frac{\sum_{i=1}^{n}\left({O}_{i}-\overline{O }\right)\left({P}_{i}-\overline{P }\right)}{\sqrt{\sum_{i=1}^{n}{\left({O}_{i}-\overline{O }\right)}^{2}}\sqrt{\sum_{i=1}^{n}{\left({P}_{i}-\overline{P }\right)}^{2}}}\right)}^{2}$$

5 $$RMSE=\sqrt{\frac{1}{n}{\sum }_{i=1}^{n}{\left({O}_{i}-{P}_{i}\right)}^{2}}$$

6 $$MAE=\frac{1}{n}{\sum }_{i=1}^{n}\left|{O}_{i}-{P}_{i}\right|$$

#### Variable relative importance evaluation

Among tree-based machine learning algorithms, sklearn provides two methods of feature selection: average impurity reduction and average precision reduction. We chose the mean decrease in impurity (MDI) to assess the relative importance of feature variables. The underlying scoring standard is Gini importance.

## Results

### Model performance

**Figure 4:**
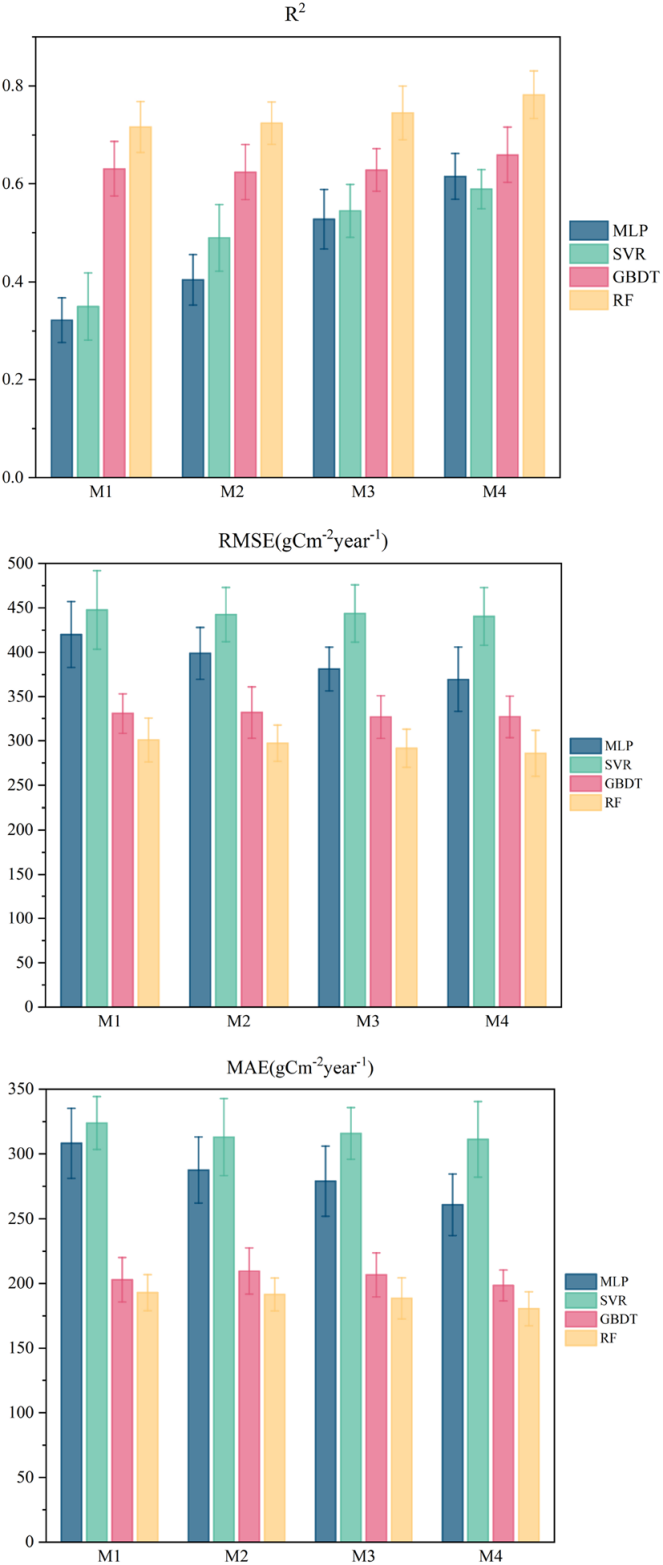
Performance of machine learning models under four training strategies.

We compared the performance of four representative machine learning models under four training modes. As shown in Fig. 4, the R^2^ values of the four models tended to increase with an increase in the environmental factors considered under the different training strategies of M1, M2, M3, and M4. By contrast, the RMSE and MAE values decreased as the environmental factors increased.

Under M1, R^2^ was lower overall than those under the other three models, and the average levels of RMSE and MAE were higher than those of the other three strategies. The reason for this phenomenon may be that the input variables of the model were too few and the environmental factors related to soil respiration were not sufficiently considered.

All four machine learning models performed best under the M4 strategy, with a higher R^2^ and lower RMSE and MAE values. Under M4, 17-dimensional input variables were considered, the variety of input data was the largest, and the model best fitted the nonlinear relationship between soil respiration and environmental factors. The performances of the four algorithms in different training modes are listed in Table 3.

**Table 3:** Performance of the model under the four strategies.

training modes	Models	R^2^	RMSE	MAE
M1	MLP	0.32207	420.09406382	308.31110335
M1	SVR	0.34977	447.68002547	323.92855055
M1	GBDT	0.63097	330.87989013	202.94027771
M1	RF	0.7165	300.77074217	192.88348008
M2	MLP	0.40413	398.90389638	287.62485300
M2	SVR	0.48962	442.42576473	313.05805901
M2	GBDT	0.62442	331.94889809	209.62937988
M2	RF	0.72435	297.27552155	191.48620076
M3	MLP	0.52767	381.22984085	279.06949794
M3	SVR	0.54464	443.77675415	315.89459784
M3	GBDT	0.62853	326.95122547	206.68278593
M3	RF	0.74514	291.56052771	188.46141941
M4	MLP	0.61558	369.53101593	260.87872089
M4	SVR	0.58924	440.41624230	311.34475190
M4	GBDT	0.65957	327.17100824	198.49270962
M4	RF	0.78216	285.89642430	180.41855427

**Figure 5:**
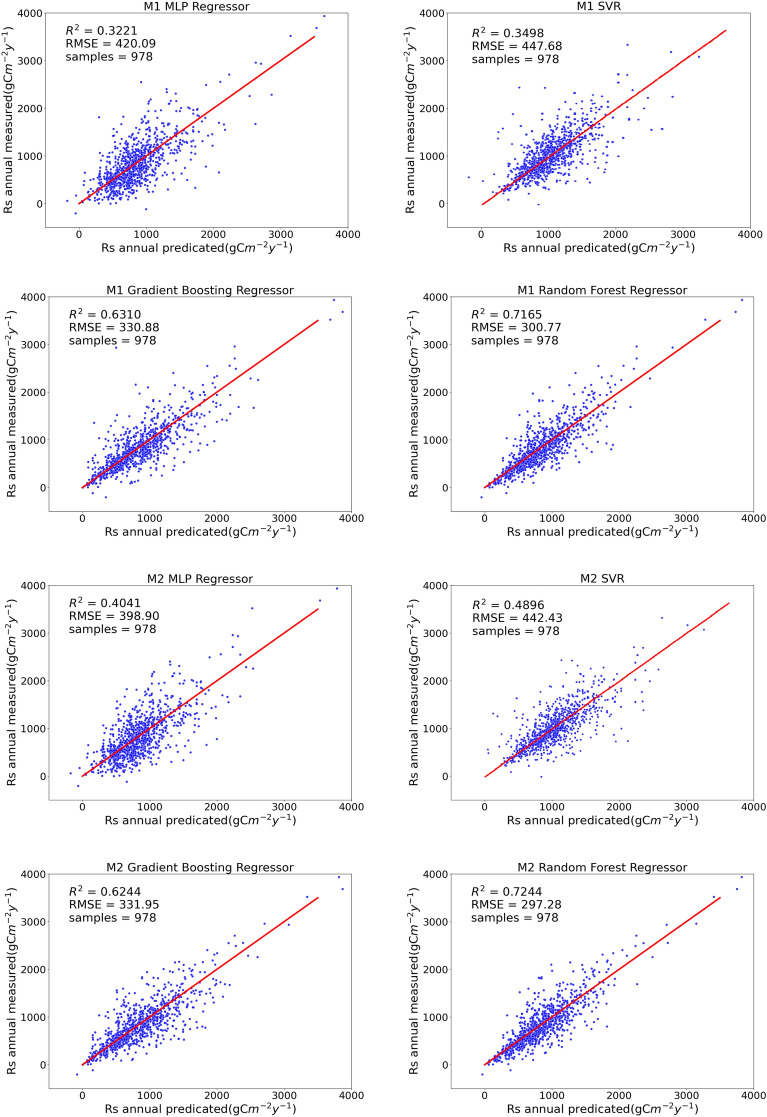

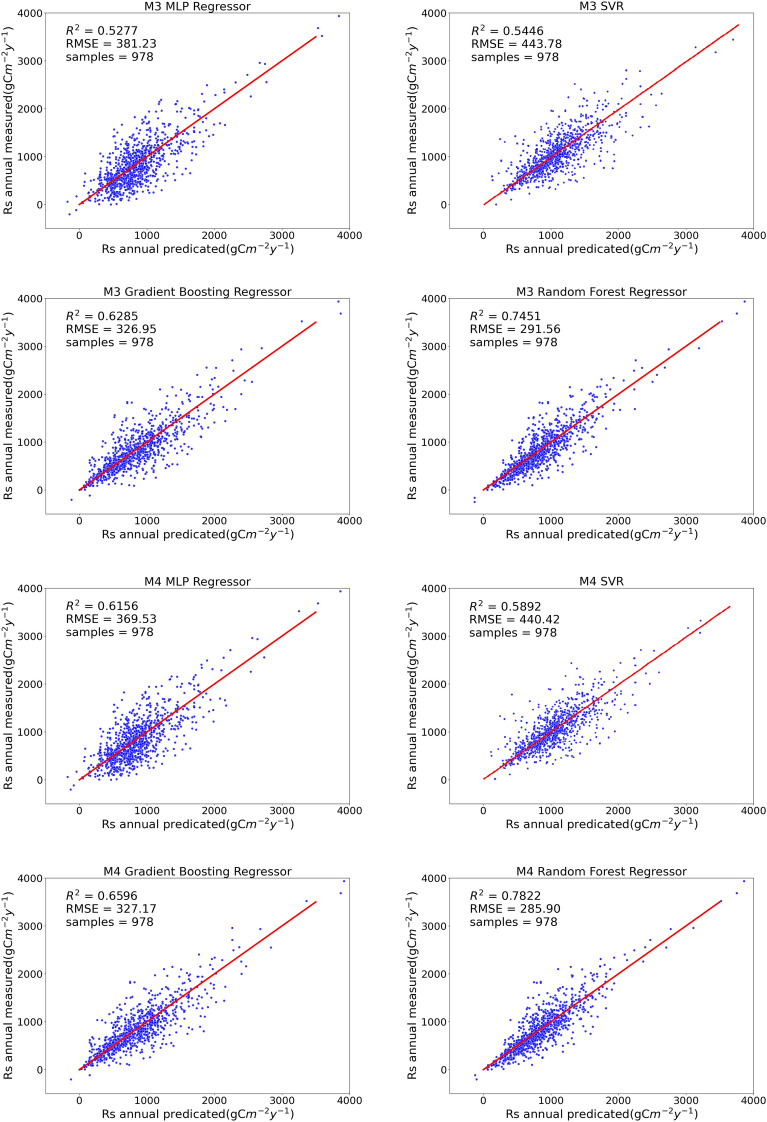
Comparison of annual measured and predicted values of soil respiration for the machine learning model under four training strategies.

Among the four machine learning models, the performance of RF was the best, followed by GBDT, and the fitting degrees of SVR and MLP were relatively low. The performance of the RF model under the M4 strategy was R^2^ = 0.78216, and the RMSE and MAE were 285.89742430 gCm^−2^ year^−1^ and 180.41855427 gCm^−2^ year^−1^, respectively. Figure 4 shows a comparison of the true and predicted values of the four models for the four training modes. A comparison of the annual measured and predicted values of soil respiration of the machine learning model under the four training strategies is shown in Fig. 5.

### Relative importance of environmental factors

Based on the previous analysis and discussion, the performance of the RF machine-learning model was better than that of the other three models. Therefore, this section discusses the relative importance of environmental variables based on the RF model.

**Figure 6:**
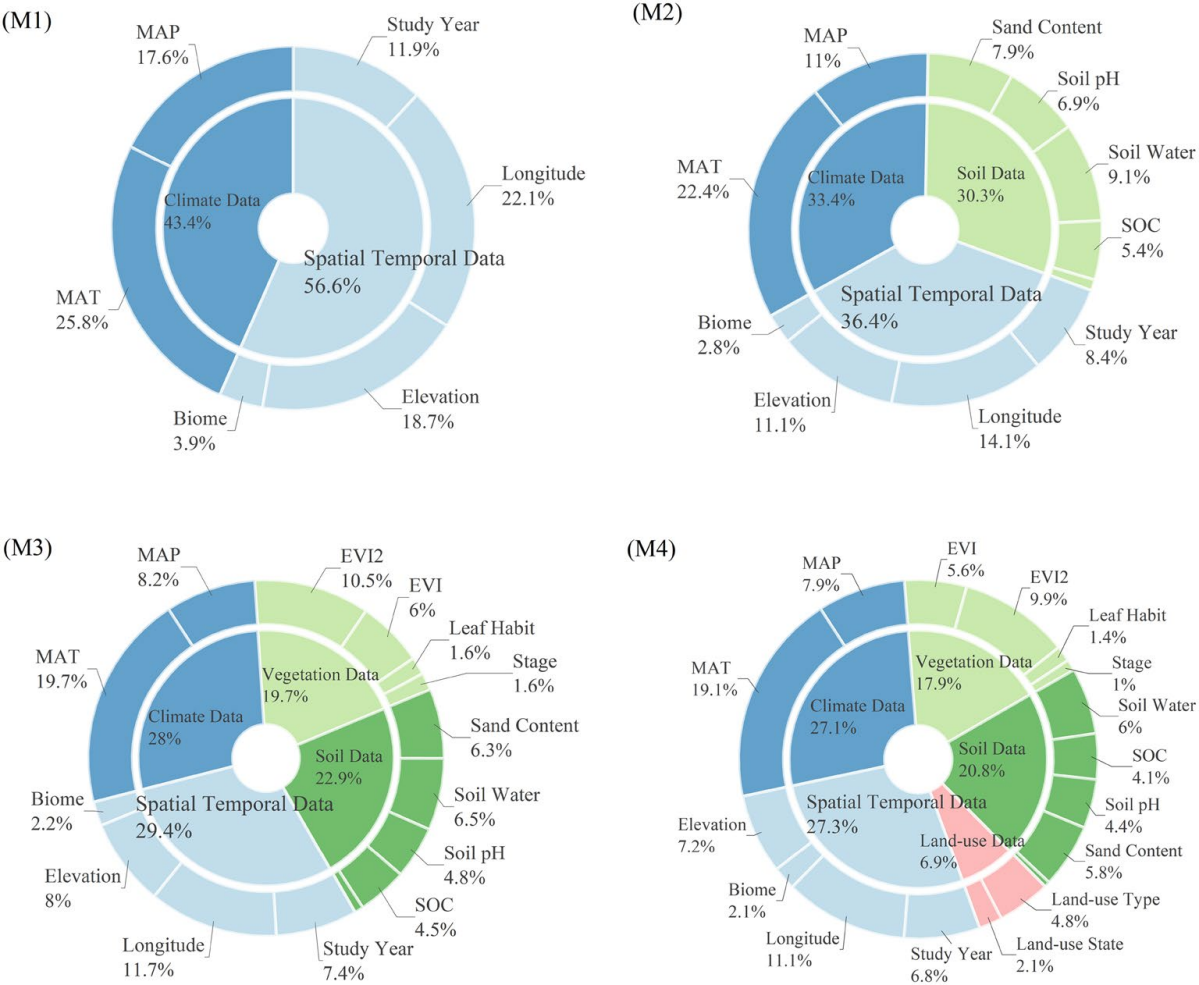
Relative importance of environmental factors under M1, M2, M3, and M4 strategies.

**Figure 7:**
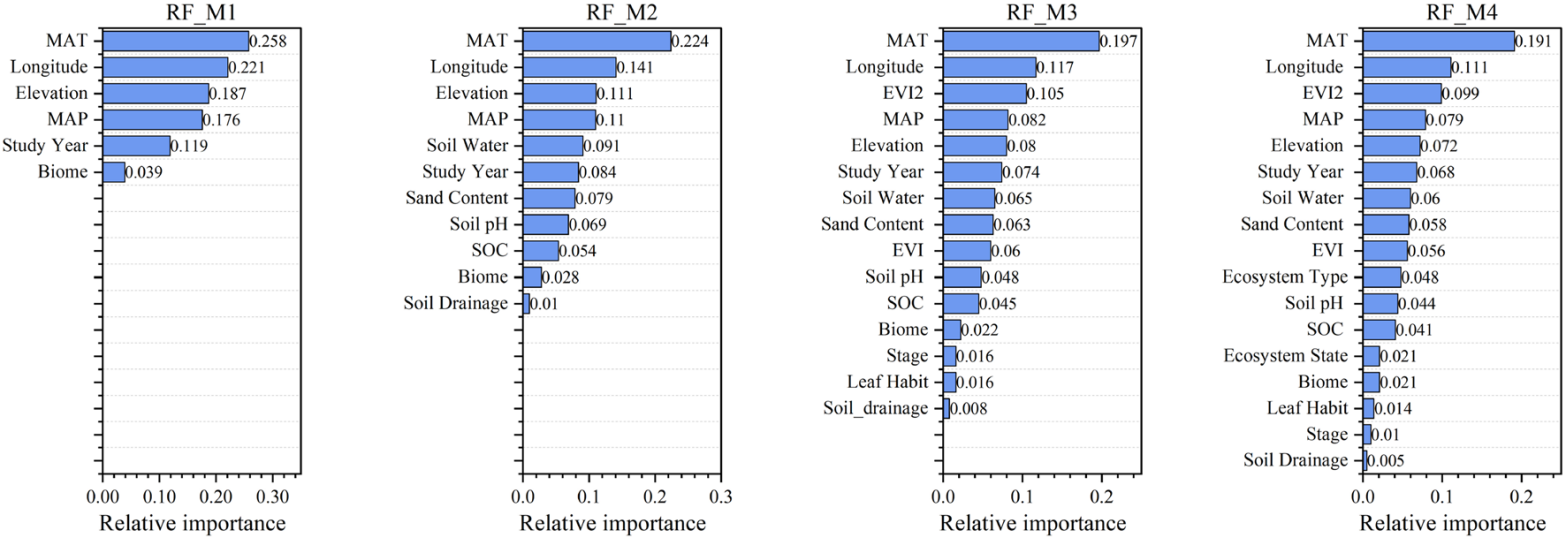
Relative importance ranking of individual environmental factors under M1, M2, M3, and M4 strategies.

We employed a feature importance method based on the mean decrease in impurity (MDI) for the environmental factor importance assessment. The contributions of environmental factors to the annual soil respiration values under strategies M1, M2, M3, and M4 are shown in Fig. 6. Figure 7 shows the relative importance of the environmental factors in descending order.

In the M1 strategy, the spatiotemporal and climatic data were considered. Although there were only two climate variables (MAT and MAP) under this strategy, they had a 38.4% impact on soil respiration. The first univariate importance was MAT, accounting for 23.9% of the variance.

The M2 strategy added 13 soil data variables to nine categories. In this model, the soil variables had a 41.5% influence on soil respiration. Among the soil variables, soil water and soil organic carbon had the most significant effects on soil respiration, with importance percentages of 8.5% and 6.6%, respectively. The importance of temperature on soil respiration decreased to 16.2% as soil environmental variables were taken into account. Other critical variables included the longitude (9.2%) and MAP (8.9%).

M3 adds remote sensing vegetation data to the previous strategy. In the remote sensing vegetation index, EVI2 was the most important factor for annual soil respiration, accounting for 7.2%. In contrast, EVI and NDVI, which have often been used in previous studies, were much less critical at 3.7% and 3.5%, respectively. This study found that EVI2 was more correlated with soil respiration. Therefore, follow-up studies should use soil respiration estimates based on the EVI2.

The M4 strategy increased the effect of land-use data on soil respiration. It can be observed that the contribution of land-use type and land-use state to the annual soil respiration value is 4.2% and 1.6%, respectively. Although there were only two land-use data variables, they contributed 5.8% to the soil respiration.

### Effects of human management or disturbance on soil respiration estimates

Human activity causes greenhouse gas (GHG) emissions and aggravate global warming. The impact of human activity on the environment has always been a research hotspot. Numerous studies have shown that the management and transformation of ecosystems by humans have a profound impact on ecosystems, but the relationship between human activities and soil respiration has rarely been studied.

This subsection categorizes the data according to the presence or absence of human management in the ecosystem and divides the data into three categories: natural, unmanaged, and managed. The RF model was used to train and estimate the relationship between environmental variables and soil respiration values under the three categories.

**Figure 8:**
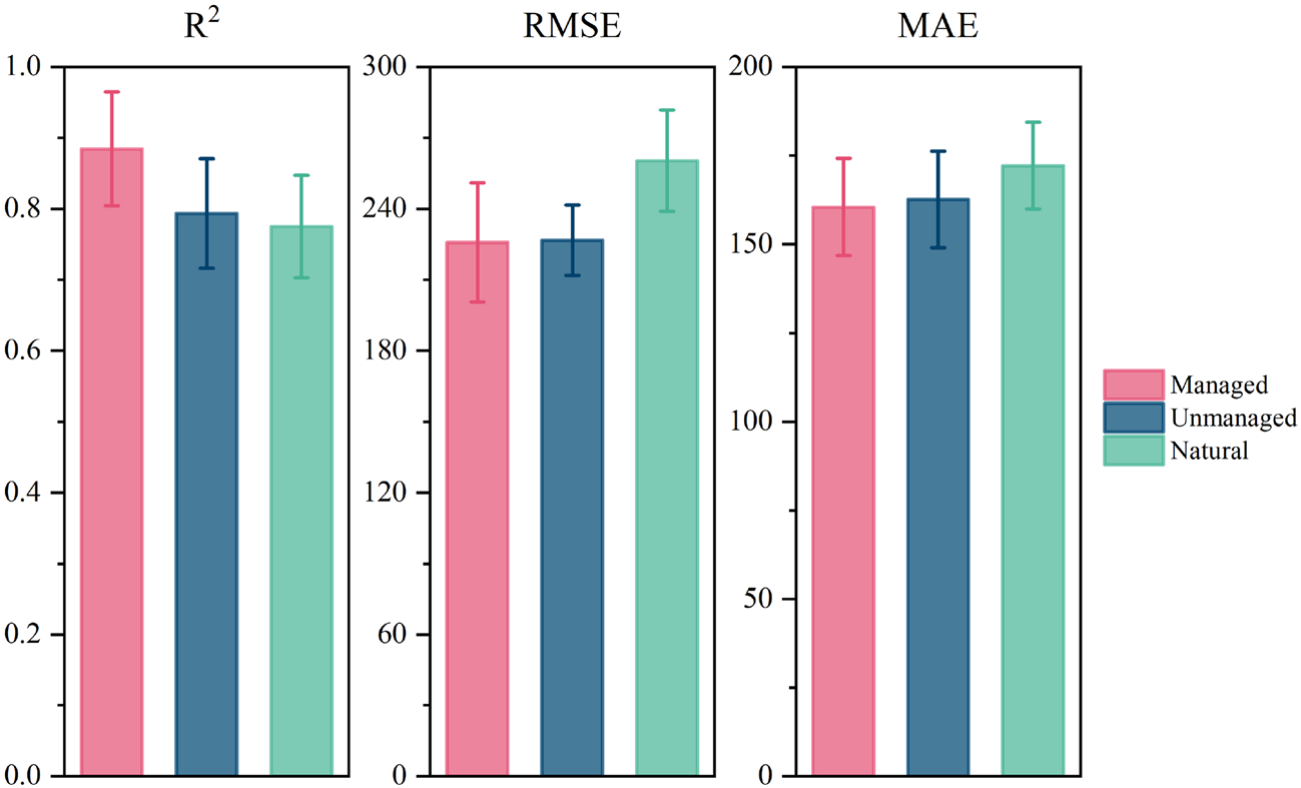
Effect of human management or disturbance on the performance of machine learning models (RF).

The model training results are shown in Fig. 8. We can clearly find that the ecosystem model with human management has a higher determination coefficient R^2^ and lower errors (RMSE and MAE), whereas the determination coefficient R^2^ of the ecosystem in the “Natural” state is relatively low, and the error (RMSE, MAE) will be higher; the ecosystem “Unmanaged”, which has been manually intervened, is somewhere between “Managed” and “Natural”. This means that when ecosystems are managed by humans, ecosystem patterns become traceable, and ecosystem heterogeneity and variability are reduced.

### Spatial distribution pattern of global soil respiration

**Figure 9:**
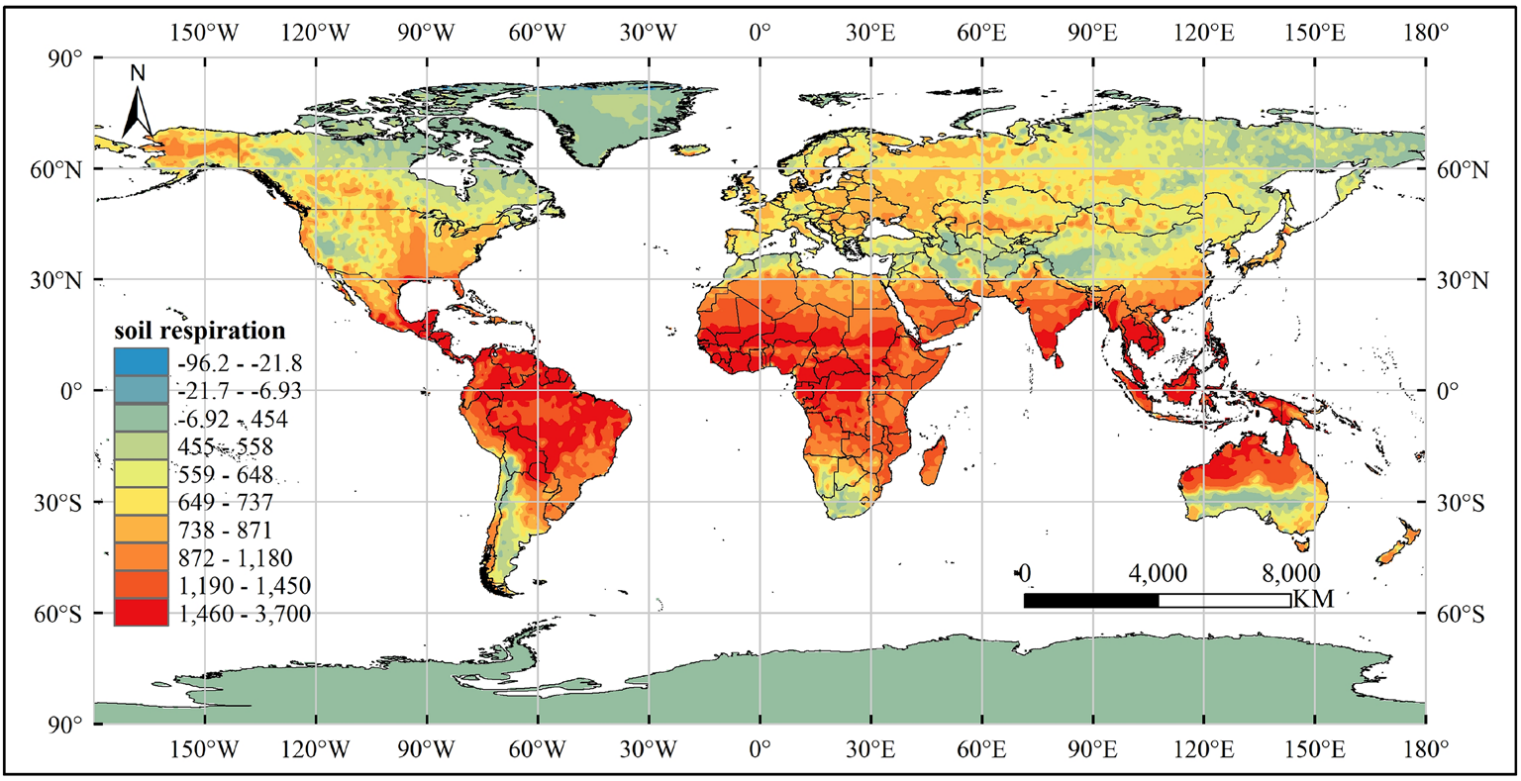
Spatial distribution pattern of global soil respiration estimated by machine learning method in 2021. The unit of the predicted soil respiration value in the figure is gCm^−2^ year^−1^. This image was created using ArcGIS, version 10.8.1, available at: https://www.arcgis.com.

We employed machine learning techniques to estimate the global soil respiration value for 2021. The random forest method, which exhibited exceptional performance in a previous study, was utilized as the modeling approach. To ensure consistency, we resampled all the driver data using interpolation to achieve a resolution of 0.5°. Subsequently, the trained RF model was applied to estimate raster data point Rs at this resolution and then scaled up to a global level. The estimated global Rs values were obtained by summing the predicted values for each pixel multiplied by its corresponding corrected surface area. The estimated results were 107 Pg C yr^−1^, R^2^ of the model was 0.78216, RMSE was 285.89742430 gCm^−2^ year^−1^, and MAE was 180.41855427 gCm^−2^ year^−1^. The spatial distribution patterns of soil respiration are shown in Fig. 9.

## Discussion

In this study, we selected four representative machine learning models and compared them to estimate soil respiration. A more ideal machine learning model was obtained. In addition, since the understanding of biotic and abiotic processes affecting soil respiration is still unclear, we selected as many as possible 27 environmental variables in five categories as input variables of the model to explore the relationship between soil respiration and environmental variables. Based on this, the global soil respiration distribution patterns and soil respiration values were estimated.

Our results show that machine learning methods can be used for large-scale soil respiration estimation problems. Random forest is a machine learning method that is more suitable for large-scale soil respiration estimation among the four typical algorithms, as shown in Fig. 4. Random forests have advantages over traditional machine learning models (MLP) for soil respiration estimation problems. Random forest can handle very high-dimensional data and has strong anti-interference and anti-overfitting abilities. Compared with support vector machines, random forests do not have the problem of kernel function selection, which affects the regression results. At the same time, RF is not as sensitive to outliers as GBDT, thus avoiding errors. Previous research has shown that RF and other deep learning models perform better than traditional machine learning models in predicting crop yields^44^, ecosystem respiration^37^, and soil organic carbon^48^. However, few studies have applied RF or other deep-learning models to estimate and predict soil respiration and its spatial distribution patterns. Our results indicate that RF models can be successfully applied to the estimation and prediction of soil respiration, and may perform better than traditional ML models, despite the relatively small datasets. In addition, with the continued increase in global carbon flux observational data and the development of deep learning methods, we expect that ML models will be more widely used to quantify the spatial distribution patterns of soil respiration and other carbon fluxes at different scales.

Because the impact of environmental factors such as spatial location, climate, soil properties, terrestrial vegetation, and land use on soil respiration is not clear enough, the impact of each category of environmental factors on soil respiration is not clear enough. Therefore, by adding one category of environmental variables at a time and observing changes in the soil respiration estimation results, we can determine the degree of influence of that category of environmental variables. This study included the environmental factors as comprehensively as possible. Five types of environmental factors and four training strategies were used to estimate the strength and distribution of soil respiration. This study aimed to clarify the effects of different environmental variables on soil respiration. In previous studies, this evaluation method was used for wheat yield, regional-scale Rs estimation, and time series estimation^30,31,44^. For example, Li et al. combined environmental factors into five strategies, used machine learning algorithms to predict wheat yield, and identified the dominant environmental factors that affect wheat yield^44^. Ebrahimi et al. classified the model input variables, added variables individually, and judged the importance of the input variables based on the degree of change in the output results after adding the variables^30^. Our results (Fig. 6) show that the impact of the five types of variables on the results from high to low is as follows: spatiotemporal data (27.3%), climate factors (27.1%), soil properties (20.8%), vegetation index (17.9%), and land use factors (6.9%). The influence of spatiotemporal data is mainly reflected in altitude, longitude, and years. The main climate factors were temperature and precipitation, with only two variables accounting for 27.1%. As shown in previous studies, temperature and precipitation have a significant impact on soil respiration^29–32^. In terms of soil property data, soil moisture, soil sand content, etc., affect soil respiration by affecting soil porosity. The larger the soil porosity, the stronger is the soil respiration. The vegetation index reflects the effect of terrestrial vegetation on soil respiration. The denser the vegetation, the stronger the respiration of the plant roots. Through the analysis of the four training strategies in this section, we can understand the impact of the five major types of environmental factors on soil respiration from a macro perspective. However, there is a shortcoming in this part of the research. The number of variables affects the proportion of the entire type. The number of variables in the five categories was different, affecting the proportion of types. The original intention of this part of the study was to explore the impacts of different categories of environmental variables on soil respiration. This inconsistent number of variables makes quantification impossible. Therefore, it is recommended to consider the impact of variables on soil respiration rather than categories.

Training strategy M4 considers all five types of environmental variables; therefore, we conduct an environmental variable importance analysis under the M4 strategy. The analysis results are shown in Figs. 6 and 7. Ranked according to the importance of environmental factors, the top five are: MAT, Longitude, EVI2, MAP and Elevation. Temperature affects almost every aspect of soil respiration. Respiration is a biochemical process that completes the glycolysis of sugar, tricarboxylic acid cycle, and electron transport chain under the action of a variety of enzymes. Research on biochemistry and physiology has demonstrated a response curve between respiration and temperature. In other words, when the temperature was low, respiration strengthened as the temperature increased. At 45–50 degrees Celsius, respiration reaches its maximum, and as the temperature increases, the respiration intensity begins to decrease^49^. The study by Ni Huang et al. showed that the temporal trend of the annual global Rs is increasing, which is mainly driven by temperature anomalies. Temperature also plays an important role in explaining interannual variations in Rs in the tropics^12^. Morgan et al. used mixed effects models and other methods to conclude that the C circulation rate decreases with increasing latitude and continuously increases with an increase in MAT and MAP^50^. Our research results show that Rs is significantly related to MAT and support the findings of Huang et al.

Longitude and latitude together constitute the geographical location of soil respiration monitoring points. In the previous analysis, it was concluded that latitude and MAT had a negative correlation; therefore, MAT was retained in the variable screening process, while latitude was removed. Our new discovery is that the effect of longitude on Rs ranks second, which is higher than what is usually thought of as a MAP. I think the possible reason is that different longitude areas have different conditions such as climate and soil conditions, biotic factors (canopy height, leaf area index and productivity), landscape patterns, natural disturbances and land use management. This results in strong spatial variability in Rs on a global scale. The results of spatial variability may be comprehensively reflected by the geographical location, that is, longitude.

We developed different machine-learning models to predict global soil respiration values. We found that the EVI2-based RF model showed slightly better performance than the NDVI- and EVI-based models in predicting the Rs values. In the feature contribution ranking based on MDI, EVI2 was 9.9%, which is much higher than EVI's 5.6%. This shows that, in terms of Rs value prediction, EVI2 has a higher contribution and can better obtain land surface vegetation information and soil respiration strength information. NDVI may be one of the most widely used vegetation indices, but it has defects such as oversaturation, susceptibility to canopy background contamination, and susceptibility to atmospheric influence, which may be the reason for the insufficient accuracy of the machine learning estimation results. In contrast, EVI overcomes the oversaturation problem of NDVI for time-series analysis and exhibits excellent performance for phenology analysis. Most previous studies have also used EVI to characterize vegetation coverage. For example, Li et al. used EVI data to predict wheat yields^44^. Warner et al. used EVI data to estimate global Rs^35^. Compared to previous studies, our new finding is that EVI2 is more representative of vegetation coverage than EVI. First, the sensors do not necessarily have blue-light bands. EVI2 can only use the near-infrared and red bands to represent the enhanced vegetation index. Therefore, EVI2 has more general applicability^46^. Second, EVI introduces the blue light band to adjust for the influence of aerosols and other atmospheric factors on the red light band. This had little effect on vegetation photosynthesis and respiration. In fact, there is a strong correlation between the visible light bands for atmospheric radiation transmission. These two reasons may make EVI2 perform better.

MAP affects soil respiration by affecting the soil moisture. It is generally believed that soil respiration is weak when MAP is low, and the soil is relatively dry and maximum when there is moderate precipitation. When precipitation is high, the soil moisture content is high, porosity decreases, soil carbon dioxide efflux is hindered, the activity of aerobic microorganisms is inhibited under anaerobic conditions, and soil respiration decreases. Huang et al. used nonlinear regression and machine learning methods to determine the relationship between soil respiration, MAP, and the evaporation index. It is positively correlated globally and is one of the main driving factors of Rs^12^. Morgan et al. explored the relationship between forest C flux and latitude, MAT, MAP, PET, VPD, solar radiation, season, etc., and further concluded that the C cycle rate is positively related to latitude and negatively related to MAT and MAP^50^. The above studies concluded that MAP is an important environmental factor that has an important impact on soil respiration.

The influence of elevation on soil respiration is often accompanied by vegetation and climate, which usually change with elevation. The higher the elevation, the lower the temperature, and the vegetation also changes gradually with the change in elevation. The highest points tended to have the lowest litter and soil respiration^51^. Studies by Kane et al. showed that the growing season and annual soil respiration are negatively correlated with elevation gradient^52^. In our results, the contribution of the elevation environmental factor was 7.2%, ranking fifth. In general, along the elevation gradient, multiple factors interact, causing soil respiration to decline with increasing altitude.

This study divides data into three categories: natural, unmanaged, and managed. The RF model was used to train and estimate the relationship between environmental variables and soil respiration values. It was concluded that the degree of fit of the land soil respiration model after human management and development was higher than that in the natural state. We find that the ecosystem model with human management has a higher determination coefficient R^2^ and lower errors (RMSE and MAE), whereas the determination coefficient R^2^ of the ecosystem in the “Natural” state is relatively low, and the error (RMSE, MAE) will be higher; the ecosystem “Unmanaged” is somewhere between “Managed” and “Natural” (Fig. 7). Humans manage and develop ecosystems in various ways, including forest harvesting, thinning, ring cutting, grassland grazing, mowing, shading, soil fertilization, and farmland cultivation. These development and management methods have an impact on ecosystems. For example, farmland tillage changes the soil permeability and moisture conditions. Tillage destroys soil aggregates, exposes organic matter, and accelerates organic matter loss. The loss of organic matter means that the substrate for soil respiration is depleted over time and soil respiration gradually weakens. After a long period of farming (artificial management), and after the ecosystem has been transformed according to human operation requirements, the originally complex interaction of factors in the ecosystem has become unified and standardized, and therefore more regular. This may explain the high R^2^ values. After land that has been cultivated for a long time is abandoned, it gradually returns to its original appearance, the loss of soil organic matter gradually recovers, the organic matter increases, and the carbon storage also increases, but there is still a gap with the natural state of land. This is probably why "Unmanaged" falls between "Natural" and "Managed”.

We used machine learning methods to estimate the global soil respiration value in 2021; the estimated value was 107 Pg C yr^−1^. Previous related studies have obtained estimates of global Rs, but these estimates vary widely. The smallest result is 72.6 Pg C yr^−1^ and the largest result is 108.6 Pg C yr^−1^, the difference ratio reaches 49.59%. There were significant differences in the soil respiration estimates. What might have caused this problem? First, the dimensions of the driving data are relatively single, which may lead to lower accuracy of the estimation results. RAICH et al. used a climate-driven regression model to estimate CO2 emissions for each month from 1980 to 1994, yielding annual soil respiration emissions of 80.4 Pg C yr^−1^^53^. This study only used climate data as the basis for estimation, and the data were relatively singular, ignoring other environmental factors that may affect Rs. Several similar studies have been conducted. For example, Hashimoto et al. used climate data to obtain global CO_2_ emission fluxes of 78 Pg C yr^−1^ and 91 Pg C yr^−1^ in different models^54,55^. Second, the accuracy of the model's expression of biotic and abiotic processes often determines the accuracy of its prediction results. For example, in CO_2_ production models, the carbon allocation problem is difficult to express. In addition, some basic relationships remain unclear and the model cannot accurately predict them. The input–output relationship established by empirical models must be supported by sufficient field measurement data. In addition, the same data input for different models may produce different results. Third, differences in data resolution may lead to large deviations in the estimation results. Adachi et al. used data with a spatial resolution of 4 km × 4 km and found that the global soil respiration in 2001 and 2009 was 94.8 and 93.8 Pg C yr^−1^, respectively^9^. Zhao et al. used an artificial neural network model to estimate the global average Rs from 1960 to 2012 as 93.3 ± 6.1 Pg C yr^−1^^56^. The spatial resolution of the resulting data is 5’ × 5’ pixels. Huang et al. used 1 × 1 km spatial resolution data and found that the average annual global RS from 2000 to 2014 was 72.6 Pg C yr^−1^^12^. Compared to previous studies, this study included environmental factors related to Rs as much as possible. To compare the performances of the four machine learning models, RF was selected to predict the global distribution pattern of Rs. Try to avoid accuracy problems caused by the data and models as much as possible.

We used a machine learning model to derive the spatial distribution pattern of global Rs in 2021. The spatial distribution patterns of soil respiration are shown in Fig. 9. The strength of soil respiration is affected by environmental factors, such as MAT, Longitude, EVI2, MAP and Elevation. In areas with relatively developed economies and obvious human activities, the soil respiration value is significantly higher than that in other regions at the same latitude, such as Europe, eastern China, and the eastern United States. Human activities may have been reflected in the vegetation index.

## Conclusion

1. Among the four machine learning models, the random forest exhibited the best performance and was the most suitable for estimating soil respiration problems.
2. Compared with ecosystems in a "natural" state, machine learning models fit better and reduce errors on ecosystems "managed" by humans.
3. In terms of the importance of environmental variables, MAT was the environmental variable with the most significant impact on soil respiration, followed by Longitude, EVI2, MAP and Elevation.
4. The EVI2 vegetation index can better reflect the vegetation coverage on the ground. Compared with NDVI and EVI, EVI2 has more vital environmental variable importance. To improve the accuracy of ecosystem estimation in follow-up research, more consideration should be given to using EVI2 as a remote-sensing vegetation index.
5. The spatial distribution pattern of global soil respiration and total value of soil respiration in 2021 were estimated.

## Data Availability

Data sets generated during the current study are available from the corresponding author on reasonable request.

## References

[1] Jenkinson DS, Adams DE, Wild A (1991). Model estimates of co2 emissions from soil in response to global warming. Nature.

[2] Post WM, Emanuel WR, Zinke PJ (1982). Soil carbon pools and world life zones. Nature.

[3] Lei J, Guo X, Zeng Y (2021). Temporal changes in global soil respiration since 1987. Nat. Commun..

[4] Rustad LE, Huntington TG, Boone RD (2000). Controls on soil respiration: implications for climate change. Biogeochemistry.

[5] Bond-Lamberty B, Bailey VL, Chen M (2018). Globally rising soil heterotrophic respiration over recent decades. Nature.

[6] Smith P (2004). Soils as carbon sinks: The global context. Soil Use Manag..

[7] Smith P (2008). Land use change and soil organic carbon dynamics. Nutr. Cycl. Agroecosyst..

[8] Lal R (2004). Soil carbon sequestration to mitigate climate change. Geoderma.

[9] Adachi M, Ito A, Yonemura S (2017). Estimation of global soil respiration by accounting for land-use changes derived from remote sensing data. J. Environ. Manag..

[10] Barba J, Cueva A, Bahn M (2018). Comparing ecosystem and soil respiration: Review and key challenges of tower-based and soil measurements. Agric. For. Meteorol..

[11] Dou X, Yang Y (2018). Estimating forest carbon fluxes using four different data-driven techniques based on long-term eddy covariance measurements: Model comparison and evaluation. Sci. Total Environ..

[12] Huang N, Wang L, Song X P, et al. Spatial and temporal variations in global soil respiration and their relationships with climate and land cover. Sci. Adv. 6(41), (2020). 10.1126/sciadv.abb8508 PMC7541079 33028522

[13] Liu J, Chen JM, Cihlar J (1997). A process-based boreal ecosystem productivity simulator using remote sensing inputs. Remote Sens. Environ..

[14] Melton JR, Arora VK (2016). Competition between plant functional types in the Canadian terrestrial ecosystem model (ctem) v. 2.0. Geosci. Model Dev..

[15] Lawrence DM, Fisher RA, Koven CD (2019). The community land model version 5: Description of new features, benchmarking, and impact of forcing uncertainty. J. Adv. Model. Earth Syst..

[16] Wang YP, Law RM, Pak B (2010). A global model of carbon, nitrogen and phosphorus cycles for the terrestrial biosphere. Biogeosciences.

[17] Yuan W, Liu D, Dong W (2014). Multiyear precipitation reduction strongly decreases carbon uptake over northern china. J. Geophys. Res. Biogeosci..

[18] Lienert S, Joos F (2018). A bayesian ensemble data assimilation to constrain model parameters and land-use carbon emissions. Biogeosciences.

[19] Smith B, Wårlind D, Arneth A (2014). Implications of incorporating n cycling and n limitations on primary production in an individual-based dynamic vegetation model. Biogeosciences.

[20] Olin S, Lindeskog M, Pugh TAM (2015). Soil carbon management in large-scale earth system modelling: Implications for crop yields and nitrogen leaching. Earth Syst. Dyn..

[21] Krinner G, Viovy N, De Noblet-Ducoudré N, et al. A dynamic global vegetation model for studies of the coupled atmosphere-biosphere system: Dvgm for coupled climate studies. Global Biogeochem. Cycles 19(1), (2005).

[22] Ito A (2019). Disequilibrium of terrestrial ecosystem co2 budget caused by disturbance-induced emissions and non-co2 carbon export flows: A global model assessment. Earth Syst. Dyn..

[23] Zeng N, Qian H, Munoz E, et al. How strong is carbon cycle-climate feedback under global warming? Geophys. Res. Lett. 31(20), (2004).

[24] Papale D, Black TA, Carvalhais N (2015). Effect of spatial sampling from European flux towers for estimating carbon and water fluxes with artificial neural networks. J. Geophys. Res. Biogeosci..

[25] Ai J, Jia G, Epstein HE (2018). MODIS-based estimates of global terrestrial ecosystem respiration. J. Geophys. Res. Biogeosci..

[26] Byrne B, Wunch D, Jones DBA (2018). Evaluating gpp and respiration estimates over northern midlatitude ecosystems using solar-induced fluorescence and atmospheric CO_2_ measurements. J. Geophys. Res. Biogeosci..

[27] Yuan W, Luo Y, Li X, et al. Redefinition and global estimation of basal ecosystem respiration rate. Glob. Biogeochem. Cycles 25(4): (2011).

[28] Jagermeyr J, Gerten D, Lucht W (2014). A high-resolution approach to estimating ecosystem respiration at continental scales using operational satellite data. Glob. Change Biol..

[29] Jian J, Bahn M, Wang C (2020). Prediction of annual soil respiration from its flux at mean annual temperature. Agric. For. Meteorol..

[30] Ebrahimi M, Sarikhani MR, Safari Sinegani AA (2019). Estimating the soil respiration under different land uses using artificial neural network and linear regression models. Catena.

[31] Hanoon MS, Ahmed AN, Zaini N (2021). Developing machine learning algorithms for meteorological temperature and humidity forecasting at Terengganu state in Malaysia. Sci. Rep..

[32] Hu J, Zhou J, Zhou G, et al. Improving estimations of spatial distribution of soil respiration using the bayesian maximum entropy algorithm and soil temperature as auxiliary data. Plos One. 11(1), (2016). 10.1371/journal.pone.0146589 PMC4726581 26807579

[33] Jian J, Frissell M, Hao D, et al. The global contribution of roots to total soil respiration. Glob. Ecol. Biogeogr. (2022).

[34] Tramontana G, Ichii K, Camps-Valls G (2015). Uncertainty analysis of gross primary production upscaling using random forests, remote sensing and eddy covariance data. Remote Sens. Environ..

[35] Warner DL, Bond-Lamberty B, Jian J (2019). Spatial predictions and associated uncertainty of annual soil respiration at the global scale. Glob. Biogeochem. Cycles.

[36] Warner DL, Guevara M, Inamdar S (2019). Upscaling soil-atmosphere co2 and ch4 fluxes across a topographically complex forested landscape. Agric. For. Meteorol..

[37] Zhu X, He H, Ma M, et al. Estimating ecosystem respiration in the grasslands of Northern China using machine learning: Model evaluation and comparison. Sustainability, 12(5), (2020).

[38] Were K, Bui DT, Dick ØB (2015). A comparative assessment of support vector regression, artificial neural networks, and random forests for predicting and mapping soil organic carbon stocks across an afromontane landscape. Ecol. Indic..

[39] Jian J, Vargas R, Anderson-Teixeira K (2021). A restructured and updated global soil respiration database (srdb-v5). Earth Syst. Sci. Data.

[40] Crippen R, Buckley S, Belz E, et al. NASADEM global elevation model: methods and progress. 2016. Pasadena, CA: Jet Propulsion Laboratory, National Aeronautics and Space Administration, 2016, (2016).

[41] Kottek M, Grieser J, Beck C (2006). World map of the köppen-geiger climate classification updated. Meteorologische Zeitschrift.

[42] Gorelick N, Hancher M, Dixon M (2017). Google earth engine: Planetary-scale geospatial analysis for everyone. Remote Sens. Environ..

[43] Muñoz-Sabater J, Dutra E, Agustí-Panareda A (2021). ERA5-land: A state-of-the-art global reanalysis dataset for land applications. Earth Syst. Sci. Data.

[44] Li L, Wang B, Feng P, et al. Developing machine learning models with multi-source environmental data to predict wheat yield in china. Comput. Electron. Agric. 194 (2022).

[45] Williams DL, Goward S, Arvidson T (2006). Landsat. Photogrammetr. Eng. Remote Sens..

[46] Jiang Z, Huete A, Didan K (2008). Development of a two-band enhanced vegetation index without a blue band. Remote Sens. Environ..

[47] Bergstra J, Bengio Y (2012). Random search for hyper-parameter optimization. J. Mach. Learn. Res..

[48] Heuvelink GBM, Angelini ME, Poggio L (2021). Machine learning in space and time for modelling soil organic carbon change. Eur. J. Soil Sci..

[49] Lloyd J, Taylor JA (1994). On the temperature dependence of soil respiration. Funct. Ecol..

[50] Banbury Morgan R, Herrmann V, Kunert N (2021). Global patterns of forest autotrophic carbon fluxes. Glob. Change Biol..

[51] Nakane K. DYNAMICS of soil organic carbon and its seasonal variation in a cool-temperate beech/fir forest on mt. odaigahara. Jpn. J. Ecol. (1978).

[52] Kane ES, Pregitzer KS, Burton AJ (2003). Soil respiration along environmental gradients in Olympic National Park. Ecosystems.

[53] Raich JW, Potter CS, Bhagawati D (2002). Interannual variability in global soil respiration, 1980–94. Glob. Change Biol..

[54] Hashimoto S, Carvalhais N, Ito A (2015). Global spatiotemporal distribution of soil respiration modeled using a global database. Biogeosciences.

[55] Hashimoto S (2012). A new estimation of global soil greenhouse gas fluxes using a simple data-oriented model. PLoS ONE.

[56] Zhao Z, Peng C, Yang Q (2017). Model prediction of biome-specific global soil respiration from 1960 to 2012. Earth’s Fut..

